# Technology Acceptance Behavior and Feedback Loop: Exploring Reverse Causality of TAM in Post-COVID-19 Scenario

**DOI:** 10.3389/fpsyg.2021.682507

**Published:** 2021-09-13

**Authors:** Erum Ishaq, Sajid Bashir, Ramsha Zakariya, Aisha Sarwar

**Affiliations:** ^1^Bahria Business School, Bahria University, Islamabad, Pakistan; ^2^Department of Business Studies, Namal Institute, Mianwali, Pakistan; ^3^Capital University of Science and Technology, Islamabad, Pakistan

**Keywords:** technology acceptance model, post-COVID-19, computer efficacy, self-regulated learning, mastery experience

## Abstract

In this study, we considered the reverse causality of the technology acceptance model, specifically in the post-COVID-19 scenario. We propose a theoretical model that considers the impact of technology acceptance behaviors after COVID-19 over the beliefs of a user in terms of perceived ease of using technology and its usefulness. More specifically, we suggested that acceptance of technology post-COVID-19 may have influenced many technology-related individual factors such as computer efficacy, mastery experience, and self-regulated learning of users, while using technology that in turn may have affected beliefs of users about ease of using technology. Such an effect is ultimately reflected in the belief of technology usefulness and favorable post-COVID-19 attitude of users toward using technology. We further extend our model to identify mastery orientation of individuals that may moderate the relationship between technology acceptance behaviors and favorable attitude toward using technology in the post-COVID-19 scenario. Both practical and theoretical implications of this perspective are discussed.

## Introduction

Of all theories, the technology acceptance model (TAM) is considered the most influential and commonly employed theory for describing the acceptance of information systems and technology of an individual. TAM, based on the theory of planned behavior (TPB) (Ajzen, 1991) and originally proposed by Davis (1986), provides an explanation of the determinants of computer acceptance of users across a broad range of end-user computing technologies and user populations (Lee et al., 2003; Marangunić and Granić, 2015).

According to TPB, the performance of a particular behavior of an individual is determined by her/his intent to perform that behavior. The intent is itself informed by attitudes toward the behavior, subjective norms about engaging in the behavior, and self-perceptions about whether the individual will be able to successfully engage in the target behavior or not. The attitudes are shaped by beliefs, the norms are developed by normative beliefs and motivation to comply, and the perceived behavioral control is informed by the beliefs of whether the individual possesses the opportunities and resources that are required to engage in the behavior. According to Ajzen (1985), an attitude toward a certain behavior is a positive or negative evaluation of performing that behavior. Deriving on the same concept as TPB, TAM is a conceptual model for technology acceptance which suggests that the actual usage of the system is a response that can be determined or predicted by user motivation, which, consequently, is directly influenced by an external stimulus. Davis (1986) moreover suggested that the motivation of a user can further be explained by the attitude of a user toward the system, which in turn is predicted by two major beliefs, perceived usefulness (PU) and perceived ease of use (PEOU). Perceived usefulness is defined as the extent to which a person believes that using a particular system would enhance her/his job performance, whereas the PEOU is defined as the degree to which a person believes that using a particular system would be effortless (Marangunić and Granić, 2015). Behavior toward technology is usually measured in terms of frequency of use, amount of time spent in using, actual number of usages, and diversity of usage (Lee et al., 2003).

Despite that today TAM is believed to be the most dominant model in investigating factors affecting acceptance of the technology of users; however, it is to be noted that the basic premise from which TAM is derived, i.e., TPB, has also been tested and supported for the possibility of reverse causality among its components (Sussman and Gifford, 2019). This demonstration of reverse-causal relations from behavior to the base components suggests that the TAM should also be taken into account for the inclusion of reciprocal causal relations. However, to date, the case of reverse causality for TAM has not been considered. The purpose of the current research is to propose reverse-causal influence among components of TAM. More specifically, we attempt to develop a theoretical model that considers the possibility of reverse-causal influences from technology acceptance behaviors back to base components of the model such as PEOU, PU, and finally attitude toward using technology. Our motivation to study these relationships comes from the post-COVID-19 situation where COVID-19 restrictions have called for inevitable and immediate adoption of technology in almost every field (Velikic et al., 2020). To address our research objectives, we relied upon prior literature concerning TAM and some of the critical insights from contemporary post-COVID-19 literature. We argue that as the world faced COVID-19 as a global pandemic, people had no choice but to accept using technology not just for performing daily tasks but also for ensuring survival (Velikic et al., 2020). This situation, where acceptance of using technology was inevitable, irrespective of the attitude of individuals toward using technology, thus called for our attention over reconsidering the causal links in TAM.

**FIGURE 1:**
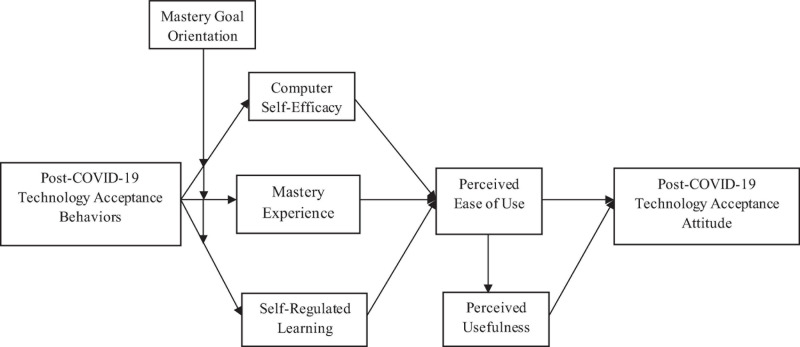
Conceptual framework.

The conventional TAM describes the technology acceptance behaviors of individuals as a processual mechanism that initiates from their attitude toward technology acceptance. We contribute toward literature by proposing that unlike conventional causal links in TAM, the attitude of individuals toward technology acceptance in the post-COVID-19 world is expected to be much more favorable than pre-COVID-19 and that is due to unprecedented acceptance of using technology by individuals in the post-COVID-19 world. This is in line with the original conception of the theory of reasoned action by Fishbein and Ajzen (1975) in which they did propose a notion of feedback loop that specifically refers to the influence of actual behavior of individuals over attitudes and subjective norms to change. Performance of a specific behavior provides new information regarding expectations, issues of control, and the probable outcomes of the behavior which ultimately results in the development of new beliefs and attitudes (Ajzen and Fishbein, 2005). We believe that since the post-COVID-19 situation has resulted in the acceptance of technology as the only means to survive and perform, such acceptance may not only have provided new but much more favorable information or feedback loop regarding control of individuals over using technology, enhanced their chances of mastery experience but also resulted in self-regulation of their technology learning. Such an impact may ultimately be reflected in the perception of individuals regarding technology ease of use and its usefulness, resulting in a much more favorable attitude among people in general toward accepting technology than before COVID-19. Proposing reverse causality for TAM, specifically concerning the post-COVID scenario, can be of critical value for practitioners while formulating business strategies as it implicates the need to capitalize on the possible favorable change in technology acceptance attitude among individuals in the post-COVID-19 world. Such conceptualization also presents a fresh perspective toward designing intervention mechanism that focuses more on mandating technology acceptance behaviors to bring about change in the attitude of users toward using technology. More specifically, in terms of technology acceptance, the concept suggests that as accepting technology becomes inevitable for users in the post-COVID scenario, not only is their self-learning compelled, but also their efficacy toward using technology is elevated due to the first-hand experience, and in addition, their chances of experiencing mastery using technology is enhanced, which ultimately may have reflected in a favorable attitude toward accepting technology in general. An enriched understanding of the concept may further enable implementing technological change in multiple domains where technology adoption is not only inevitable but is also required on an immediate basis as in the case of the post-COVID scenario. Proposed conceptual framework is presented in Figure 1.

## Technology Acceptance Model and Consideration of Reverse Causality

Ever since the inception of this model, one distinctive feature of TAM studies has been to attempt model extension with external variables of PEOU and PU which include individual, organizational, and task characteristics. Some external variables were introduced into TAM as suggested by several researchers. For example, Venkatesh and Davis (2000) define the external variables of PU, such as social influence (subjective norms) and cognitive instruments (job relevance, image, quality, and result demonstrability). Similarly, Venkatesh (2000) provides the external variables of PEOU, such as anchor (computer self-efficacy, perceptions of external control, computer anxiety, and computer playfulness) and adjustments (perceived enjoyment and objective usability).

However, despite the rapid rise of technology, particularly information and communication technologies, and its incorporation into the private and professional life of users, a decision regarding its acceptance or rejection remained optional for individuals till we faced the pandemic. We seek to explain how the feedback loop of accepting technology without having an option in the post-COVID-19 situation can have an impact on the beliefs of users regarding PU and PEOU, thus ultimately shifting their attitude toward using technology. More specifically, we identify computer self-efficacy, mastery experience, and self-regulated learning (SRL) of individuals as factors to explain the causal relationship between technology acceptance behaviors and change in perception of ease of using technology. We believe that such an impact is ultimately reflected in PU of technology and post-COVID-19 attitude of users toward technology.

## Computer Self-Efficacy

Control has an evident effect on key dependent variables such as intention and behavior in a wide range of domains. Consistent with TPB, in the area of information system research, PEOU has been seen as a determinant of attitude that drives acceptance behavior, while control is considered to be a key predictor of PEOU (Venkatesh, 2000). However, exploring the reverse causality effects in current research concerning TPB, we attempt to depart from the basic framework of TAM and propose a reverse-causal relationship in which technology acceptance behaviors may provide complementary information regarding control over using the technology of individuals, thus leading to favorable perception of ease toward using technology.

In the domain of technology, internal control is conceptualized as computer self-efficacy, an individual difference variable representing the belief of an individual about her/his ability to perform a specific task/job using a computer (Compeau and Higgins, 1995). Literature suggests that practicing a certain behavior on frequently or repeatedly performing a specific task is often the strongest factor influencing percept of the efficacy of an individual (Joo et al., 2000). Likewise, with an increasing direct behavioral experience with technology, individuals get the chance to assess their skills personally. In doing so, they are not only able to overcome biasness toward their ability to use technology but it also reduces their fear toward using technology. This leads to enhanced efficacy toward using technology over time (Igbaria and Iivari, 1995).

The extent to which information technology was used for performing day-to-day official tasks post-COVID-19 is unprecedented. People were bound to use technology without any options if they were to survive their lives in terms of careers. The need for survival demands acceptance and performance of behaviors unconditionally or without any excuse (McLeod, 2007). Keeping in view, we propose that with enhanced dependency over technology and thus its acceptance to perform day-to-day tasks and work roles, judgments of individuals about confidence in using technology post-COVID-19 is a reflection of their specific, concrete, and unbiased attributes toward their ability to use technology. These specific attributes are based on information derived from direct personal experience with technology systems. We also believe that dependency and frequent interaction with the technology of individuals in the post-COVID-19 world may have reduced their technology phobia to a greater extent and probably shifted them to technophile from fearful. This can lead to much-enhanced efficacy levels of individuals in the post-COVID-19 world than otherwise. Keeping in view that confidence or efficacy of users in their technology-related abilities and knowledge serve as the basis for judgment of an individual about how easy or difficult technology will be to use (Venkatesh, 2000), thus we propose the following:

**Proposition 1: *Computer self-efficacy may mediate the relationship between post-COVID-19 technology acceptance behaviors and post-COVID-19 perceived technology ease of use***

## Mastery Experience

Mastery experience refers to personal experience of success (Bandura, 1977). The post-COVID-19 situation required accepting technology as the only way to perform day-to-day tasks and to achieve desired work goals. Thus while accepting technology behaviors post-COVID-19, individuals had to fully concentrate on the use of technology for performing the task at hand. Hence, they had to be fully engaged in using technology if they were to achieve their minimum performance standards. The literature suggests that a situation in which attention is fully directed toward an activity or individuals are completely absorbed or engaged in an activity may lead to having an enhanced probability of achieving mastery experience (Yeh et al., 2019). We believe that since accepting technology was the only option available for performing day-to-day activities and work roles in the post-COVID-19 scenario, thus level of seriousness and focus for learning the use of technology of users was much higher than otherwise. This can lead to having a higher probability of experiencing mastery concerning the use of technology in the post-COVID-19 scenario.

A high level of mastery experience by users may provide favorable feedback about accepting technology, thus changing rigid, inaccurate, or unfavorably biased judgment regarding ease of using technology to much stable and accurate unbiased and favorable judgment by post-COVID-19 users of technology. Indirect support for this is also suggested by Bandura (1982) whereby mastery experiences (personal attainments) are viewed as providing the strongest information for the formation of control judgments. Such judgments may be more stable and accurate, compared to judgments developed by the indirect information provided by modeling, persuasion, and arousal (Gist and Mitchell, 1992). Since control judgments are also central to developing perception about ease of using technology, thus we may say that a high probability of experiencing mastery after acceptance of using technology in the post-COVID-19 situation may lead to developing favorable ease of using technology perception among users.

**Proposition 2: *Mastery experience in using technology may mediate the relationship between post-COVID-19 technology acceptance behaviors and post-COVID-19 perceived technology ease of use***

## Self-Regulated Learning

Self-regulation is a distinctive feature of social cognitive theory and is defined as a set of principles and practices by which people monitor their own behaviors and consciously adjust those behaviors in pursuit of personal goals. SRL is thus a proactive way of learning in which people manage their own learning processes (Butler and Winne, 1995; Wan et al., 2012). Self-regulation of an individual toward learning can be influenced to a great extent by differences in the situations under which individuals are operating (Wan et al., 2012). Self-regulated learners set goals for their learning and then attempt to control their cognition and motivation, guided and constrained by their goals and the contextual features in the environment (Pilling-Cormick and Garrison, 2007; Puzziferro, 2008).

As mentioned earlier, the post-COVID-19 scenario required an immediate shift toward using technology for performing routine tasks and work-related tasks. Such immediate post-COVID-19 shift toward adopting the usage of technology required more proactive and self-regulated involvement on the part of an individual to develop and upgrade their technology competence due to limited support functions in place. The majority of the work-related tasks in the post-COVID-19 scenario were to be performed online while working from home where workers did not physically attend a formal office environment and hence did not have the opportunity to interact face-to-face with their supervisors (Barnes, 2020; Naciri et al., 2020). Learners in such situations have to structure the time, pace, and strategy of their own learning processes (Puzziferro, 2008).

Self-regulated learning process involves proactive information seeking on part of learners, continuous adjustments, and finding solutions when they encounter difficulty (Wan et al., 2012), thus in the post-COVID-19 scenario, it was much needed and the only most viable strategy for learning the use of technology. Considering that deploying SRL involves greater initiative/responsibility on part of the learner and thus higher levels of motivation, chances of successful performance and satisfaction with using technology for conducting different tasks may also be much higher among users post-COVID-19 than otherwise (Wang et al., 2013). Such an effect may ultimately be reflected in a favorable post-COVID-19 perception of ease of using technology among users. Thus we propose that:

**Proposition 3: *Self-regulated learning in using technology may mediate the relationship between post-COVID-19 technology acceptance behaviors and post-COVID-19 perceived technology ease of use***

## Mastery Goal Orientation

Orientation is the most common characteristic of goals associated with learning. In general, high achievement goals direct the behavior of individuals toward continuously striving to accomplish these goals. Individuals with mastery goal orientations seemingly exert consistent efforts and hard work to enhance their intellectual abilities (Wan et al., 2012; Ishaq et al., 2019). Mastery-learning-oriented individuals are intrinsically motivated and are less susceptible to failure, because their sense of satisfaction with the work is not driven by external reasons (Nicholls, 1984; Valentini and Rudisill, 2006). In the mind of mastery-learning-oriented individuals, an effort is a more significant factor than ability on the way to achieving success. When individuals set and target their own learning goals and find motivation from within to progress toward those goals, they are more likely to stay resilient through difficult learning situations and often find the learning process more gratifying (Zumbrunn et al., 2011). It is further suggested in the literature that when people experience their behavior as self-determined by intrinsic motivation, they develop both mastery and efficacy (Yeh and Lin, 2018). Post-COVID-19 situation required individuals to strive to develop technology-related competence on an immediate basis to achieve both daily tasks and meet work performance standards. We thus believe that in such challenging situation individuals with mastery goal orientation may have shown more resiliencies to adjust better and learn to use technology in a much efficient and effective manner than others. Such an impact is ultimately reflected in a much more favorable perception of post-COVID-19 ease of using technology for people having mastery orientations than for people who lack such orientations.

**Proposition 4: *Mastery goal orientations may moderate the mediated relationship between post-COVID-19 technology acceptance behaviors and post-COVID-19 perceived technology ease of use through computer self-efficacy such that mediated relationship is stronger when mastery orientation of individuals is high than low***

**Proposition 5: *Mastery goal orientations may moderate the mediate relationship between post-COVID-19 technology acceptance behaviors and post-COVID-19 perceived technology ease of use through mastery experience such that mediated relationship is stronger when mastery orientation of individuals is high than low***

As mentioned earlier that a mastery goal orientation pertains to developing abilities, mastering new skills, accomplishing challenges, and developing alternative strategies when working on difficult tasks of an individual (Ishaq et al., 2019). Thus individuals with high learning goal orientation have a more positive attitude toward learning. They set goals for intrinsic reasons such as improving their level of understanding and learning or taking on a particular challenge. Accordingly, they are likely to believe that they can control and regulate their own learning process by using a variety of personal SRL strategies. Previous research has shown that learning goal orientation has a positive influence on the use of SRL of individuals (Yeh and Lin, 2018). We thus propose that once accepted for use as a result of the COVID-19 situation, individuals having mastery orientations will be able to self-regulate their learning strategies toward using technology better than individuals with low mastery orientations. This may also enable such individuals to achieve and perform their tasks better, thus resulting in much favorable ease of use perception of technology than individuals having low mastery orientations.

**Proposition 6*: Mastery goal orientations may moderate the mediated relationship between post-COVID-19 technology acceptance behaviors and post-COVID-19 perceived technology ease of use through self-regulated learning such that mediated relationship is stronger when individuals mastery orientation is high than low***

## Post-COVID-19 Technology Acceptance Behaviors and Change in Post-COVID-19 Technology Perceived Usefulness and Post-COVID-19 Attitude Toward Technology Among Users

There is extensive empirical evidence accumulated over a decade that PEOU is a key direct determinant of PU (e.g., Venkatesh, 1999; Venkatesh and Davis, 2000). It is suggested that the less effortful a system or technology is to use, the more usage of it can increase the performance of users, thus enhancing their perception of technology usefulness. Particularly in the post-COVID-19 situation where a shift to using technology was inevitable for individuals in every field, thus individuals who were not even much used to or familiar with using technology in performing day-to-day tasks or work roles had adopted its usage. This new familiarity with using technology in carrying out routine tasks and performing work roles may not only have changed their rigid unfavorable notions regarding technology usage but also made them realize its other benefits such as convenience, ease of communication, cost-effectiveness, and most importantly surviving through as challenging situations as COVID-19. Since attitude toward technology is the reflection of how technology is perceived in terms of ease and usefulness, thus we propose that the favorable feedback loop or information regarding the use of technology in the post-COVID-19 situation may have positively influenced computer efficacy, mastery experience, and SRL of users that further derives favorable postpandemic attitude toward technology acceptance *via* PEOU and PU. Also, people with mastery orientations may have adapted to a post-COVID-19 technology-dependent environment better, thus resulting in a comparatively favorable postpandemic attitude toward technology than others.

**Proposition 7: *Post-COVID-19 technology acceptance behaviors, computer efficacy, mastery experience, and self-regulated learning may have a positive influence over the post-COVID-19 attitude toward technology via post-COVID-19 perceived ease of use and usefulness***

**Proposition 8: *Individuals mastery orientations, may moderate the relationship between post-COVID-19 technology acceptance behaviors, computer efficacy, mastery experience, self-regulated learning, and post-COVID-19 attitude toward technology via post-COVID-19 perceived ease of use and usefulness such that mediated relationship is stronger when individuals mastery orientation is high than low***

## Discussion

Technology plays a crucial role in performing day-to-day tasks and work roles in the modern world of today. However, before COVID-19, the use of technology was not inevitable, rather it was a matter of choice to adopt technology or to continue using conventional methods for accomplishing routine tasks and work roles as per the convenience of individuals or organizations, especially in underdeveloped countries. Thus, opting for technology still remained a big question for many to date. As the world faced COVID-19 as a global pandemic, people had no choice apart from accepting technology for use in almost every field ranging from work, education, health care, and commerce to even performing routine tasks (Velikic et al., 2020). This unprecedented acceptance of technology usage post-COVID-19 thus allowed us to consider the probability of reverse causality in TAM. We attempt to contribute toward literature by studying the mechanism through which post-COVID-19 acceptance of technology use travels back to developing a comparably favorable attitude toward using technology in the post-COVID-19 world. In line with past literature that suggests the possibility of reverse-causal relationships in TPB (Sussman and Gifford, 2019), a concept on which TAM is based, current research is the only attempt in the literature to consider the possibility of reverse causality in TAM. More specifically, we suggest that the post-COVID-19 acceptance of technology behaviors will influence computer efficacy, mastery experience, and SRL of users while using technology that will have a favorable impact on the beliefs of users concerning ease of using technology. Such an effect will ultimately be reflected in perceived technology usefulness and favorable post-COVID-19 attitude of users toward using technology. We also contribute toward literature by proposing a contingency framework to study reverse causality in TAM specifically in the post-COVID-19 scenario. More specifically, we propose that the goal orientations of an individual may also play a key role in deciding that how much favorable impact the post-COVID-19 situation may have over developing technology acceptance attitude among individuals.

Such propositions are crucial for not just technology researchers but the business community at large, since it allows researchers to understand the change in the attitude of individuals toward the technology in the post-COVID-19 situation and reflect it in their future research areas and formulation of business strategies to capitalize on such change. The validity of such propositions is more in those regions where the availability of technology and its use for performing daily tasks was still not very high, such as in the case of many developing and underdeveloped countries (Barnes, 2020). Technology shift must have jumped by leaps and bounds in those countries as compared to more advanced countries. Businesses with overseas operations expanding to such regions, thus must account for this change in attitude toward technology and capitalize on such change.

Despite having several strengths, we do acknowledge that the current study is limited in terms of empirical validation of its propositions. However, we believe that the current study opens avenues for technology researchers to understand past research findings of TAM with a different perspective, identify possible novel research topics, and conduct future empirical studies to validate the reverse-causal links in TAM. It is also recommended for future researchers to further investigate reverse causality in TAM through considering a variety of other possible individuals and their situational factors such as ambiguity tolerance, role of leadership in this crisis, family support, etc. Future researchers also need to consider the impact of change in post-COVID-19 technology attitude over business future strategies and business digital transformations in the post-COVID-19 world.

## Data Availability Statement

The original contributions presented in the study are included in the article/supplementary material, further inquiries can be directed to the corresponding author.

## Author Contributions

All authors listed have made a substantial, direct and intellectual contribution to the work, and approved it for publication.

## Conflict of Interest

The authors declare that the research was conducted in the absence of any commercial or financial relationships that could be construed as a potential conflict of interest.

## Publisher’s Note

All claims expressed in this article are solely those of the authors and do not necessarily represent those of their affiliated organizations, or those of the publisher, the editors and the reviewers. Any product that may be evaluated in this article, or claim that may be made by its manufacturer, is not guaranteed or endorsed by the publisher.
